# Perioperative leukocyte–plateletcrit shift as a prognostic signature in glioblastoma

**DOI:** 10.1007/s11060-025-05302-8

**Published:** 2025-10-20

**Authors:** Petr Krupa, Filip Kotek, Marketa Krupova, Simona Paulikova, Petra Kasparova, Tomas Cesak

**Affiliations:** 1 Department of Neurosurgery, Faculty of Medicine in Hradec Kralove, Charles University, University Hospital Hradec Kralove, Hradec Kralove, Czech Republic; 2 The Fingerland Department of Pathology, Faculty of Medicine in Hradec Kralove, Charles University, University Hospital Hradec Kralove, Hradec Kralove, Czech Republic; 3 Department of Oncology and Radiotherapy, University Hospital Hradec Kralove, Sokolska 581, Hradec Kralove, 50005 Czech Republic

**Keywords:** Glioblastoma, Leukocytes, Platelets, Plateletcrit, Inflammation, Survival, Cox model, Temozolomide

## Abstract

**Purpose:**

Circulating inflammatory indices derived from routine blood counts may offer pragmatic prognostic information in glioblastoma (GB), yet the prognostic role of plateletcrit (PCT) and of peri-treatment dynamics in leukocyte–platelet coupling remains underexplored.

**Methods:**

We retrospectively studied 95 adults with histologically confirmed GB (48 men, 47 women; median age 64.5 years) treated adjuvantly with radiotherapy and chemotherapy with complete blood counts obtained at four windows: pre-operative, post-operative, pre-adjuvant, and post-adjuvant. From leukocytes, platelets (PLT), plateletcrit (PCT), and mean platelet volume (MPV) we derived all within-timepoint ratios and inter-timepoint differences (Δ). Overall survival (OS) in days was modeled using Cox proportional hazards (per + 1 SD), with Benjamini–Hochberg false-discovery summaries; a multivariable model adjusted for age, sex, extent of resection, radiotherapy, concomitant temozolomide (TMZ), and number of adjuvant TMZ cycles was calculated.

**Results:**

Median overall survival (OS) was 406 days, with 80 deaths. In univariate models, dynamic indices predominated: the post-operative rise in leukocytes relative to plateletcrit (Δ post-op − pre-op leu/PCT) showed the strongest adverse association (HR 1.60, 95% CI 1.22–2.10; *p* = 0.0007; BH–FDR q = 0.09), with concordant signals for Δ leu/PLT (HR 1.43, 95% CI 1.14–1.79; *p* = 0.002) and a protective inverse for Δ PCT/leu (HR 0.66, 95% CI 0.50–0.87; *p* = 0.003). Cross-sectionally, higher post-operative leu/PCT and leu/PLT and higher post-adjuvant leukocytes and leu/MPV were adverse (all *p* < 0.05). In the multivariable model adjusting for age, sex, extent of resection, radiotherapy, concomitant temozolomide (TMZ), and number of adjuvant TMZ cycles, Δ leu/PCT remained independently associated with worse OS (HR 1.62, 95% CI 1.04–2.52; *p* = 0.031), while concomitant TMZ (HR 0.18, 95% CI 0.05–0.68; *p* = 0.012) and greater adjuvant TMZ exposure (per + 1 SD; HR 0.48, 95% CI 0.29–0.78; *p* = 0.003) were protective.

**Conclusions:**

Dynamic leukocyte–platelet coupling—especially Δ (post-op − pre-op) leu/PCT—provides independent prognostic information beyond standard covariates. CBC-based trajectories are low-cost and scalable and warrant prospective validation. Interpretation is limited by the absence of systematic MGMT methylation data and requires external validation and comparison with other prognostic scoring systems.

**Supplementary Information:**

The online version contains supplementary material available at 10.1007/s11060-025-05302-8.

## Introduction

Glioblastoma (GB) remains the most common and aggressive primary malignant brain tumor in adults, with median overall survival near 14–15 months despite maximal safe resection and temozolomide-based chemoradiation [1]. Routine complete blood count (CBC)–derived inflammatory indices have drawn interest as pragmatic prognostic markers, with multiple studies and meta-analyses linking neutrophil-to-lymphocyte ratio (NLR), platelet-to-lymphocyte ratio (PLR), and related composites to poorer survival in glioma and GB [2–4]. Emerging work further suggests that temporal dynamics in these ratios—rather than single preoperative snapshots—may carry stronger prognostic information after standard chemoradiation [5].

Beyond lymphocyte-normalized indices, growing evidence implicates the platelet compartment in glioma biology through platelet–tumor crosstalk, pro-angiogenic signalling, and treatment-related shifts, with higher or rising platelet measures often associating with worse outcomes [6]. These observations motivate platelet-centric metrics that capture not only counts but also the effective platelet mass and activation state.

Plateletcrit (PCT)—the fraction of whole blood volume occupied by platelets—is an underused, volumetric platelet index reported on many hematology analyzers; conceptually analogous to hematocrit, it integrates information from platelet count and mean platelet volume (MPV) [7, 8]. Outside neuro-oncology, higher preoperative or baseline PCT has been linked to inferior outcomes in several solid tumors, supporting its biological plausibility as a prognostic signal [9, 10]. In GB specifically, platelet-focused markers are beginning to emerge: the MPV-to-platelet count ratio (MPV/PC) has been associated with progression and survival, and studies of PLR in GB also report adverse associations, albeit with heterogeneity across cohorts [11, 12].

What remains insufficiently defined is whether leukocyte–platelet coupling that incorporates platelet mass—particularly leukocyte-to-PCT—and, critically, its peri-treatment trajectory, provides independent prognostic information in GB. We therefore quantified cross-sectional values and inter-timepoint changes (Δ) in leukocyte- and platelet-derived indices across pre-operative, post-operative, pre-adjuvant, and post-adjuvant windows, with a prespecified focus on PCT, and tested associations with overall survival in Cox models adjusted for standard covariates. We hypothesized that peri-operative Δ leu/PCT would be associated with OS beyond static, single-timepoint measures.This study addresses that gap by quantifying both cross-sectional values and inter-timepoint changes in leukocyte- and platelet-derived indices, with particular focus on PCT [8].

### Study design, setting, and ethics

This was a single-center retrospective cohort of consecutive adults with histologically confirmed GB treated at Department of Neurosurgery, University Hospital Hradec Kralove, Czech Republic between 1st of January 2018 and 31st of December 2023. Data were abstracted from electronic records and laboratory systems. The study was approved by Local Ethics committee (No. 202509 P03) and conducted in accordance with the Declaration of Helsinki.

### Eligibility, exclusions, and treatment

**Fig. 1:**
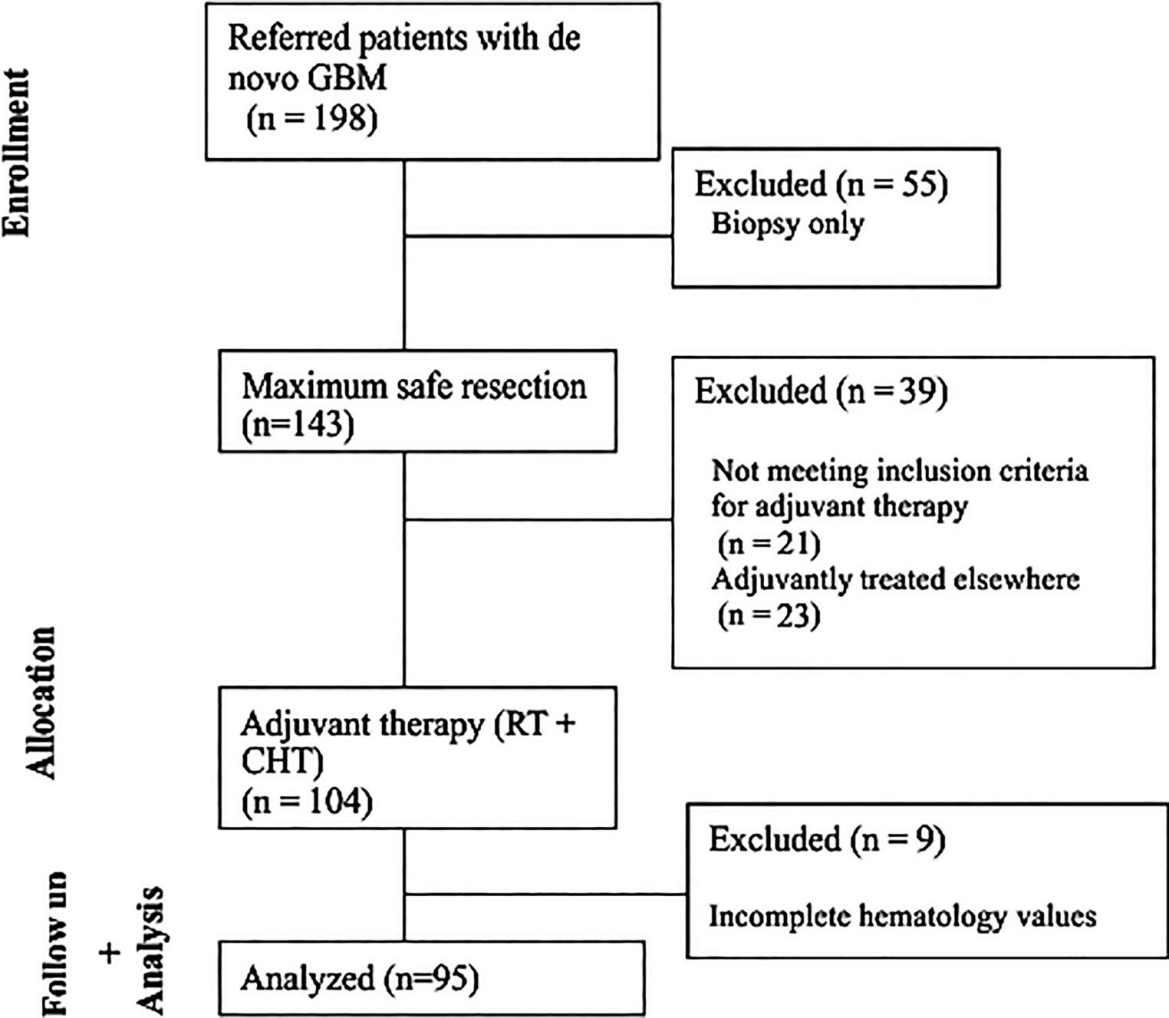
CONSORT style flowchart of patients in the study

Inclusion criteria were age ≥ 18 years, de-novo GB IDH wild-type by WHO 2021 criteria, index resection and adjuvant therapy at our center, and availability of hematology panels at predefined windows (Fig. 1). From the study were excluded patients who were not eligible for adjuvant treatment after the resection because of poor performance status. Standard therapy comprised surgery aiming for maximal safe resection, followed by radiotherapy (planned total dose 60/45 Gy in 30/15 fractions) with concomitant TMZ (75 mg/m² daily) and adjuvant TMZ 150–200 mg/m² D1–5 q28d, per local Stupp-based protocol. Imaging surveillance intervals followed service standards every 3 months at regular oncological check-ups.

### Timepoints, laboratory variables, and derived indices

Hemograms were abstracted at four windows: pre-operative, early post-operative (24 h post-surgery), pre-adjuvant, and post-adjuvant. Variables included leukocytes (×10⁹/L), PLT (×10⁹/L), MPV (fL), and PCT (%, e.g. 0.28%); MPV-to-leukocyte ratio (MPVLR) was recorded. Subsequently we computed within-timepoint ratios among leukocytes, PLT, PCT, MPV (e.g., leu/PCT, leu/PLT, PCT/leu, MPV/PLT) and inter-timepoint differences (Δ) for each absolute marker and each ratio for post-op − pre-op, pre-adj − post-op, post-adj − pre-adj, and post-adj − pre-op. For all ratio calculations involving PCT (e.g., leu/PCT, PCT/leu) we treat PCT as a fraction; therefore PCT (%) is converted to a fraction by dividing by 100 before ratio computation (e.g., 0.28% → 0.0028).

### Clinical covariates and endpoints

Clinical variables included sex, age at operation, pre-operative tumor volume, and extent of resection (EOR). EOR was classified as gross total (GTR), near-total (NTR), subtotal (STR), or partial resection (PR) on early post-op contrast-enhanced MRI according to Karschnia [13]. Moreover, Karnofsky Performance Status (KPS) and comorbidities were noted. The primary endpoint was overall survival (OS) in days, defined from operation date to death; patients without recorded survival duration were right-censored on August 26, 2025.

### Statistical analysis

We summarized OS via Kaplan–Meier and reported the median with 95% CI. Screening log-rank tests after median splits were run but the primary analyses used Cox proportional hazards models with predictors entered as z-standardized continuous variables (HR per + 1 SD). To control multiplicity during broad univariate screening, we computed Benjamini–Hochberg q-values. To reduce multiplicity, hypotheses were pre-grouped a priori into Δ-metrics and cross-sectional metrics at the four windows, and BH-FDR was applied within each family. In total, we tested N_XS = 64 cross-sectional hypotheses and N_Δ = 96 Δ-hypotheses (N_total = 160). Continuous predictors were analyzed per + 1 SD to standardize effect scale. We built a multivariable Cox model adjusted for age, sex, EOR, radiotherapy, concomitant TMZ, and number of adjuvant TMZ cycles; to limit collinearity across related Δ-ratios we excluded one of any pair with |r|>0.80 from entering together.

Within each Δ-family (e.g., post-operative − pre-operative), we computed pairwise Spearman correlations among all Δ-values and Δ-ratios. Pairs with |rₛ| ≥ 0.80 were deemed highly collinear; for such sets we retained a single representative based on clinical interpretability and univariate strength. In the leukocyte–platelet Δ-family, Δ leu/PCT showed high positive correlation with Δ leu/PLT and inverse correlation with Δ PCT/leu (|rₛ|≥0.80; Supplementary Table S1); thus Δ leu/PCT entered the multivariable model, while Δ leu/PLT and Δ PCT/leu were excluded.

Proportional hazards were assessed using Schoenfeld residuals versus log-time. Δ leu/PCT met the PH assumption, whereas adjuvant TMZ cycles and post-adjuvant leukocytes showed possible deviations (*p* < 0.05). As a sensitivity analysis, we administratively censored follow-up at 12 months and re-fit the model (see Results). We repeated all models excluding transfused patients. We quantified discrimination using Harrell’s C-index and performed bootstrap optimism correction (B = 300 resamples, patients sampled with replacement, model re-fit in each bootstrap; seed = 20251002). We report apparent and optimism-corrected C-indices in Results.

To assess incremental prognostic value, we compared a baseline clinical Cox model (age, sex, extent of resection, radiotherapy, concomitant temozolomide, number of adjuvant TMZ cycles, post-adjuvant leukocytes) to an augmented model additionally including Δ post-operative − pre-operative leu/PCT. Continuous predictors were z-standardized; ties were handled by Breslow; analyses were conducted on the same complete-case set. We report log-likelihood, AIC, BIC, likelihood-ratio tests, and Harrell’s C-index (apparent and bootstrap optimism-corrected, B = 300).

Analyses used Python with Breslow ties; significance was two-sided at α = 0.05.

## Results

### Patient population, complications and survival

We included 95 adults with GB and complete hemograms (48 men, 47 women; median age 64.5 years, IQR 56.0–71.8). The extent of resection was GTR 49, NTR 28, STR 9, PR 9. There were 80 deaths; Kaplan–Meier median overall survival (OS) was 406 days (approx. 13.3 months), and median follow-up among survivors was 945 days. No post-operative infections or major complications occurred within 24 h of surgery. In the same window, 3 patients received RBC transfusion and 1 patient received platelet transfusion. During follow-up, we observed one reoperation for intracerebral hemorrhage (post-op day 5), two subdural hygromas (days 5 and 183), and two surgical-site infections (days 22 and 355).

**Table 1:** Baseline characteristics of the study cohort

Characteristic	Value	Percent / IQR
Patients	95	
Female, n (%)	47	49.5
Male, n (%)	48	50.5
Age at surgery, median (IQR), y	64.5	56.0–71.8
Pre-op tumor volume, median (IQR), cm³	18.0	12.0–40.0
Extent of resection – GTR, n (%)	49	51.6
Extent of resection – NTR, n (%)	28	29.5
Extent of resection – STR, n (%)	9	9.5
Extent of resection – PR, n (%)	9	9.5
Deaths (events), n	80	
Censored, n	15	
Median OS, days	406	
Median follow-up among survivors, days	945	
Concomitant TMZ – yes, n (%)	64	67.4
Concomitant TMZ – no, n (%)	31	32.6
Adjuvant TMZ cycles, median (IQR)	2	0–6
Radiotherapy 60 Gy/30fr	66	69.5
Radiotherapy 45 Gy/15fr	29	30.5
Preoperative KPS	70	60–90
Postoperative KPS	70	60–90

Demographics, tumor metrics, treatment exposure, and survival summary for 95 adults with glioblastoma (48 men, 47 women). Data are shown as n (%) or median (IQR). Extent of resection (EOR) was categorized as gross total (GTR), near-total (NTR), subtotal (STR), or partial resection (PR) per early postoperative MRI. Kaplan–Meier median overall survival (OS) is reported in days; patients without recorded survival times were right-censored on August 26, 2025 (operation date → censoring date). Abbreviations: EOR = extent of resection; GTR = gross total resection; NTR = near-total resection; STR = subtotal resection; PR = partial resection; RT = radiotherapy; TMZ = temozolomide; OS = overall survival; KPS = Karnofsky Performance status; IQR = interquartile range

### Peri-treatment trajectories of hematology values

**Fig. 2:**
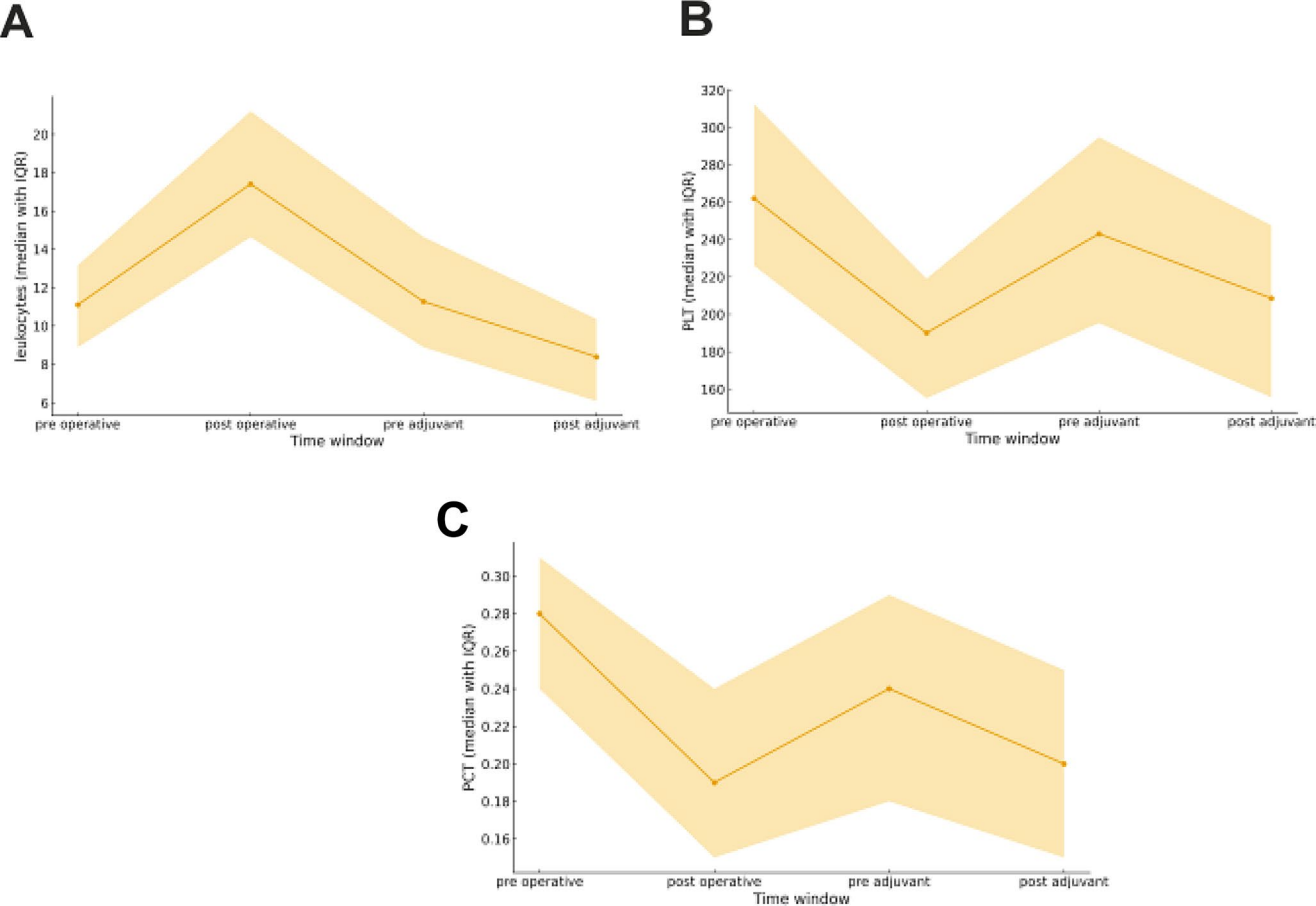
Median (IQR) trajectories of leukocytes (**A**), platelets (**B**), and plateletcrit (**C**) across pre-op, post-op, pre-adj, and post-adj windows

In descriptive analyses across the four windows, leukocyte counts rose sharply from 11.1 × 10⁹/L pre-operatively to 17.4 × 10⁹/L post-operatively, then declined toward 11.27 × 10⁹/L pre-adjuvant and 8.40 × 10⁹/L post-adjuvant. Platelet measures showed the reciprocal pattern: PLT fell from 262 × 10⁹/L pre-op to 190 × 10⁹/L post-op, with a partial rebound to 243 × 10⁹/L pre-adjuvant and 208.5 × 10⁹/L post-adjuvant, while PCT decreased from 0.275% to 0.190% post-op, recovering to 0.240% pre-adjuvant and 0.200% post-adjuvant. MPV varied modestly (10.3 fL pre-op, 10.4 fL post-op, 9.8 fL pre-/post-adjuvant), and the composite MPVLR tracked these shifts (0.893 pre-op → 0.603 post-op → 0.830 pre-adjuvant → 1.172 post-adjuvant). (Table 2; Fig. 2)

**Table 2:** Median (IQR) laboratory values at each clinical window and median (IQR) inter-timepoint changes (Δ)

marker	pre-operative	post-operative	pre-adjuvant	post-adjuvant	Δ post-operative - pre-operative	Δ pre-adjuvant - post-operative	Δ post-adjuvant - pre-adjuvant	Δ post-adjuvant - pre-operative
MPV	10.30 (9.43–10.97)	10.40 (9.65–11.00)	9.80 (9.28–10.40)	9.80 (9.10–10.53)	0.00 (-0.40–0.30)	-0.50 (-0.90–-0.20)	-0.10 (-0.40–0.20)	-0.60 (-1.10–0.10)
PLT	262.00 (226.00–312.50)	190.00 (155.00–219.00)	243.00 (195.25–294.75)	208.50 (155.50–247.50)	-64.00 (-102.00–-34.00)	54.00 (21.75–91.50)	-34.50 (-83.50–0.25)	-59.00 (-105.00–-12.00)
PCT	0.28 (0.24–0.31)	0.19 (0.15–0.24)	0.24 (0.18–0.29)	0.20 (0.15–0.25)	-0.06 (-0.11–-0.04)	0.04 (0.01–0.08)	-0.03 (-0.08–0.00)	-0.07 (-0.13–-0.00)
leukocytes	11.10 (8.88–13.16)	17.40 (14.63–21.20)	11.27 (8.89–14.64)	8.39 (6.08–10.36)	5.65 (2.28–8.82)	-5.59 (-9.88–-2.15)	-3.05 (-5.82–-0.81)	-2.81 (-5.54–-0.34)
MPVLR	0.89 (0.75–1.12)	0.60 (0.48–0.72)	0.83 (0.68–1.14)	1.17 (0.94–1.68)	-0.29 (-0.55–-0.10)	0.21 (0.09–0.45)	0.36 (0.10–0.56)	0.32 (-0.05–0.59)

For MPV, PLT, PCT, leukocytes, and MPVLR, medians (IQR) are reported at pre-operative, post-operative, pre-adjuvant, and post-adjuvant windows, together with medians (IQR) of Δ for post-op − pre-op, pre-adj − post-op, post-adj − pre-adj, and post-adj − pre-op. Units: MPV fL; PLT ×10⁹/L; PCT %; leukocytes ×10⁹/L; MPVLR unitless

Abbreviations: MPV = mean platelet volume (fL); PLT = platelets (×10⁹/L); PCT = plateletcrit (%); leu = leukocytes (×10⁹/L); MPVLR = MPV-to-leukocyte ratio; SD = standard deviation; CI = confidence interval; BH = Benjamini–Hochberg

For ratio-based indices, we observed a marked post-operative spike in leukocyte-weighted measures that partially normalized by the adjuvant phases: leu/PLT rose from 0.040 pre-operatively to 0.091 post-operatively (median Δ + 0.043), easing to 0.050 pre-adjuvant and 0.038 post-adjuvant. Likewise, leu/PCT increased from 4248 to 8791 post-operatively, then declined toward 5112 pre-adjuvant and 4133 post-adjuvant, while the inverse PCT/leu fell from 0.000235 to 0.000114 post-operatively (median Δ − 0.000098) before rebounding to 0.000196 and 0.000242, respectively. A similar perioperative surge with subsequent attenuation was seen for leu/MPV (1.11 → 1.66 → 1.21 → 0.85) and a modest, opposite shift for MPV/PLT (median Δ post-op − pre-op + 0.0168). (Table 3)

**Table 3:** Median (IQR) of leukocyte–platelet ratios and median (IQR) of their inter-timepoint changes (Δ)

ratio	pre-operative	post-operative	pre-adjuvant	post-adjuvant	Δ post-operative - pre-operative	Δ pre-adjuvant-post-operative	Δ post-adjuvant - pre-adjuvant	Δ post-adjuvant - pre-operative
MPV/PCT	3824 (3209–4343)	5333 (4538–6493)	4167 (3374–5177)	4889 (4025–6464)	1346 (645–2245)	-1149 (-1916–-377)	667 (-083–1491)	825 (036–1974)
MPV/PLT	0.04 (0.03–0.05)	0.06 (0.04–0.07)	0.04 (0.03–0.05)	0.05 (0.04–0.06)	0.02 (0.01–0.02)	-0.01 (-0.02–-0.01)	0.01 (-0.00–0.01)	0.01 (0.00–0.02)
MPV/leu	0.90 (0.76–1.12)	0.60 (0.48–0.72)	0.83 (0.68–1.14)	1.17 (0.94–1.68)	-0.29 (-0.55–-0.10)	0.21 (0.09–0.45)	0.36 (0.10–0.56)	0.30 (-0.05–0.55)
MPVLR	0.89 (0.75–1.12)	0.60 (0.48–0.72)	0.83 (0.68–1.14)	1.17 (0.94–1.68)	-0.29 (-0.55–-0.10)	0.21 (0.09–0.45)	0.36 (0.10–0.56)	0.32 (-0.05–0.59)
PCT/MPV	0.0003 (0.0002–0.0003)	0.0002 (0.0002–0.0002)	0.0002 (0.0002–0.0003)	0.0002 (0.0002–0.0002)	-0.0001 (-0.0001-0.00)	0.0001 (0.00–0.0001)	-0.000 (-0.0001-0.000)	-0.0001 (-0.0001-0.000)
PCT/leu	0.0002 (0.0002–0.0003)	0.0001 (0.0001–0.0001)	0.0002 (0.0002–0.0003)	0.0002 (0.0002–0.0003)	-0.0001 (-0.0002-0.0001)	0.0001 (0.00–0.0001)	0.00 (-0.00–0.0001)	0.00 (-0.0001–0.0001)
PLT/MPV	26.34 (21.03–32.25)	17.57 (15.05–22.28)	24.90 (20.18–30.51)	21.52 (15.55–26.38)	-6.99 (-11.86–-3.48)	6.28 (2.33–10.99)	-3.21 (-8.25–0.65)	-4.85 (-10.11–-0.13)
PLT/PCT	95,872 (91242–103727)	96,667 (90612–104006)	101,613 (97269–107958)	102,500 (94700–110278)	907 (-2469–3794)	4842 (1833–9286)	919 (-2876–4206)	6694 (2003–12663)
PLT/leu	24.72 (18.46–30.46)	10.99 (8.33–13.55)	20.14 (16.86–27.49)	26.22 (18.22–34.23)	-12.99 (-19.62–-6.77)	9.36 (5.49–15.23)	2.76 (-2.86–9.44)	0.73 (-6.54–10.00)
leu/MPV	1.11 (0.89–1.32)	1.66 (1.39–2.07)	1.21 (0.88–1.48)	0.85 (0.59–1.06)	0.53 (0.21–0.84)	-0.48 (-0.82–-0.17)	-0.31 (-0.57–-0.07)	-0.27 (-0.49–0.03)
leu/PCT	4248 (3402–5844)	8791 (7197–11065)	5112 (3761–5994)	4133 (3058–5673)	3876 (3231–5335)	-3622 (-5547–-2390)	-629 (-1939–483)	-132 (-1333–1294)
leu/PLT	0.04 (0.03–0.05)	0.09 (0.07–0.12)	0.05 (0.04–0.06)	0.04 (0.03–0.05)	0.04 (0.03–0.07)	-0.04 (-0.06–-0.03)	-0.01 (-0.02–0.00)	-0.00 (-0.01–0.01)

All pairwise ratios among MPV, PLT, PCT, and leukocytes (e.g., leu/PCT, leu/PLT, PCT/leu, MPV/PLT) and MPVLR are summarized at each time window, with corresponding medians (IQR) of Δ for post-op − pre-op, pre-adj − post-op, post-adj − pre-adj, and post-adj − pre-op. Ratios are unitless, For all ratios involving PCT, PCT (%) was converted to a fraction (e.g., 0.28% → 0.0028) prior to calculation

### Univariate analyses of values, ratios, and Δ

**Fig. 3:**
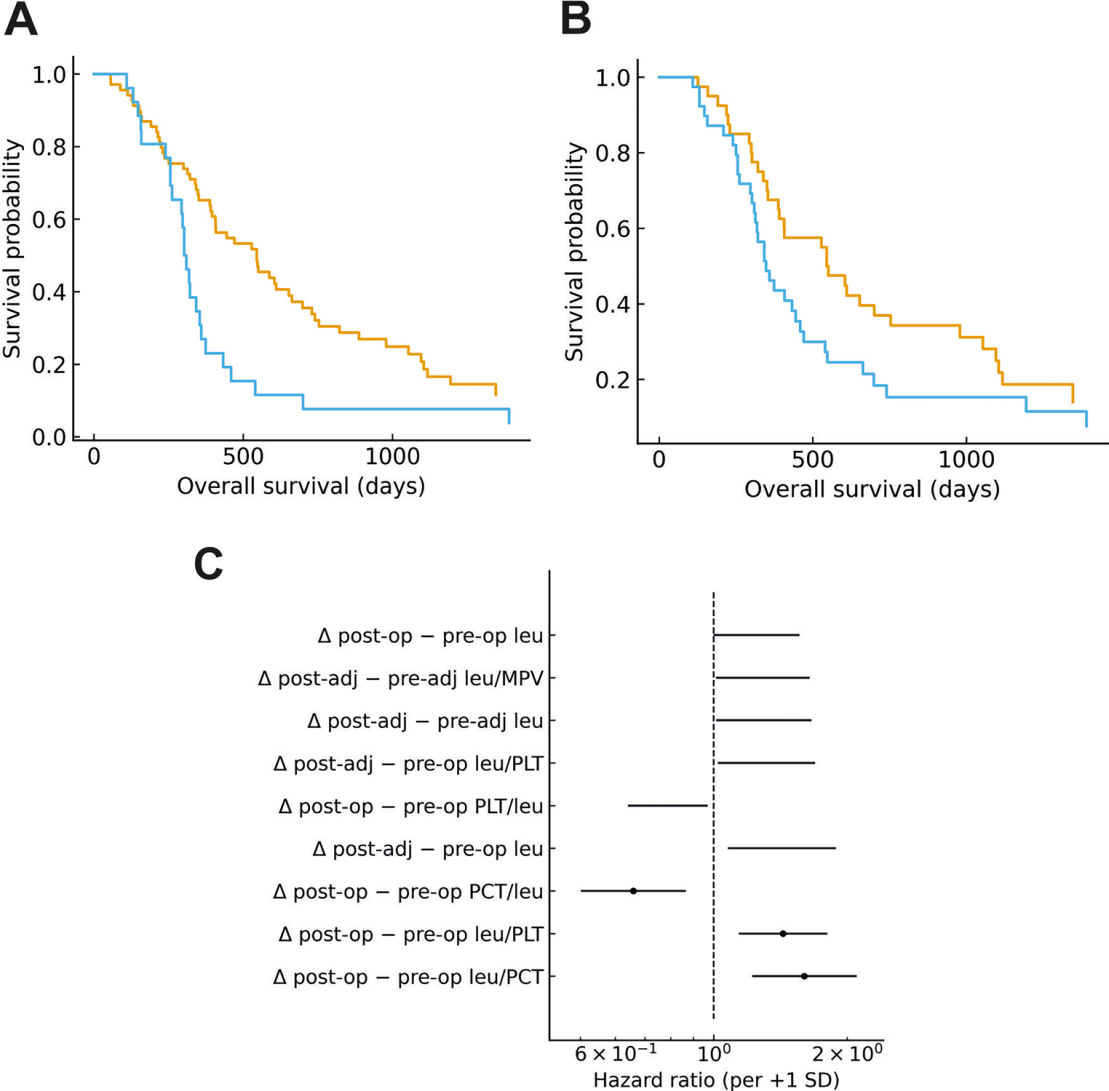
Perioperative leukocyte–platelet dynamics and overall survival in GB. (**A**) Kaplan–Meier curves stratified by the median of Δ (post-operative − pre-operative) leukocyte-to-plateletcrit ratio (leu/PCT). Patients above the median had shorter OS (two-sided log-rank *p* = 0.004). (**B**) Kaplan–Meier curves stratified by the median of Δ (post-operative − pre-operative) leukocyte-to-platelet count ratio (leu/PLT), showing a borderline separation (log-rank *p* = 0.06). KM is provided for visualization; inference is based on continuous per-SD Cox models. (**C**) Forest plot of univariate Cox proportional hazards for inter-timepoint Δ metrics (hazard ratios per + 1 SD with 95% CIs). Time origin is the date of resection. All Δ metrics were computed from complete four-window CBC data (*N* = 95). Abbreviations: leu = leukocytes; PLT = platelets; PCT = plateletcrit; OS = overall survival. Panel selection corresponds to the strongest adverse Δ signal (leu/PCT) and a concordant leukocyte-weighted index (leu/PLT), with the full Δ screen summarized in panel C. Reported p-values in panels A–B are from log-rank tests; Cox models in panel C use z-standardized predictors (per + 1 SD)

Median-split log-rank tests at single time points were not significant. In per-SD univariate Cox models, dynamic (Δ) measures predominated: the post-operative rise in leukocytes relative to plateletcrit was the strongest adverse signal (Δ post-op − pre-op leu/PCT: HR 1.60, 95% CI 1.22–2.10; *p* = 0.0007; BH–FDR q = 0.09). Concordant effects were seen for Δ leu/PLT (HR 1.43, 95% CI 1.14–1.79; *p* = 0.002), whereas the inverse Δ PCT/leu was protective (HR 0.66, 95% CI 0.50–0.87; *p* = 0.003). Across the full course, post-adjuvant − pre-operative leukocytes were also adverse (HR 1.43; *p* = 0.013). Cross-sectionally, higher post-operative leu/PCT (HR 1.33, 95% CI 1.06–1.66; *p* = 0.013), post-operative leu/PLT (HR 1.28, 95% CI 1.04–1.59; *p* = 0.022), post-adjuvant leukocytes (HR 1.34, 95% CI 1.03–1.74; *p* = 0.028), and post-adjuvant leu/MPV (HR 1.33, 95% CI 1.03–1.72; *p* = 0.029) were each associated with worse overall survival. (Fig. 3).

### Multivariable model

In the multivariable Cox model adjusting for age, sex, extent of resection, radiotherapy, concomitant temozolomide (TMZ), and the number of adjuvant TMZ cycles, the Δ post-operative − pre-operative leu/PCT remained independently prognostic (HR 1.62, 95% CI 1.04–2.52; *p* = 0.031). Concomitant TMZ was associated with lower hazard (HR 0.18, 95% CI 0.05–0.68; *p* = 0.012), and greater adjuvant TMZ exposure per + 1 SD conferred additional protection (HR 0.48, 95% CI 0.29–0.78; *p* = 0.003). Model fit was adequate (log-likelihood − 93.65; AIC 207.3; BIC 225.4). Schoenfeld residual diagnostics indicated possible time-varying effects for adjuvant TMZ cycles (*p* = 0.037) and post-adjuvant leukocytes (*p* = 0.026), whereas Δ leu/PCT showed no evidence of non-proportionality.

The adjusted Cox model achieved a Harrell’s C-index of 0.80 (apparent), with optimism-corrected C-index 0.76 from bootstrap internal validation (B = 300). Sensitivity to non-proportionality. With administrative censoring at 12 months, the effect of Δ post-operative − pre-operative leu/PCT remained directionally consistent (HR 1.31, 95% CI 0.70–2.44; *p* = 0.400), supporting the robustness of the primary Δ-association despite reduced events.

To assess the incremental value of Δ leu/PCT we compared with the baseline clinical model, adding Δ post-operative − pre-operative leu/PCT improved fit (LR χ²(1) = 4.38, *p* = 0.036), reduced AIC (207.20→204.82) and BIC (219.84→219.28), and modestly increased Harrell’s C-index (0.798→0.803 apparent; both optimism-corrected 0.763, B = 300). In the augmented model, Δ leu/PCT remained independently associated with worse OS (HR 1.58, 95% CI 1.03–2.44; *p* = 0.037). In a prespecified sensitivity analysis excluding transfused patients, Δ (post-op − pre-op) leu/PCT remained independently associated with shorter OS.

Although Δ leu/PCT satisfied the proportional-hazards assumption, adjuvant TMZ cycles and post-adjuvant leukocytes showed possible non-proportionality in Schoenfeld diagnostics. While our 12-month sensitivity analysis yielded similar inferences for the primary Δ-predictor, formal time-varying coefficient models (counting-process formulation) could further refine estimates for these covariates and represent a direction for future work. Discrimination was internally validated via bootstrap optimism correction; external validation in an independent cohort was not performed.

## Discussion

In this single-center cohort, dynamic leukocyte–platelet coupling—most notably the postoperative increase in leukocytes relative to plateletcrit (Δ leu/PCT)—was independently associated with inferior overall survival (OS), supporting the broader concept that peri-treatment inflammatory trajectories carry prognostic information in glioblastoma (GB). Our findings align with and extend prior work on CBC-derived indices in glioma, where higher neutrophil-to-lymphocyte and platelet-to-lymphocyte ratios have repeatedly correlated with worse outcomes, while recent studies emphasize that changes after Stupp chemoradiation can outperform static baselines. [4, 5, 14, 15]

The biological plausibility of leukocyte–platelet composites in GB is strong given expanding evidence that platelets actively modulate tumor biology, including angiogenesis, immune evasion, and treatment resistance via tumor–platelet crosstalk and “tumor-educated platelets” [16, 17]. Platelet-centric literature across oncology further supports the clinical relevance of platelet measures, as elevated or rising platelet indices have been linked to adverse survival in several solid tumors and are increasingly explored as dynamic biomarkers alongside immuno-inflammatory ratios [18, 19].

Our focus on plateletcrit (PCT) adds a volumetric dimension that traditional platelet counts do not capture, because PCT reflects the fraction of blood volume occupied by platelets and is mathematically derived from platelet count and mean platelet volume [8, 20]. Outside neuro-oncology, higher preoperative PCT has been associated with inferior survival—for example, in non-small cell lung cancer—supporting its candidacy as a pragmatic biomarker that integrates both platelet number and size [9]. To our knowledge, leukocyte-to-PCT and its peri-operative delta have not been systematically evaluated in GB, and our data suggest this coupling captures clinically meaningful shifts in the inflammatory–thrombocytic axis with prognostic consequences.

The signal we observed dovetails with emerging meta-analytic evidence that systemic inflammatory ratios (e.g., NLR, PLR) carry prognostic information in glioma/GB, while work centered on post-therapy values and dynamics highlights the added value of measuring trajectories rather than single snapshots [4, 5, 14]. Mechanistically, platelets can shield circulating tumor cells, regulate endothelial adhesion and extravasation, and sculpt an immunosuppressive microenvironment—processes that provide a plausible framework for why platelet mass–aware indices such as PCT, and leukocyte–PCT ratios, might track risk as patients transition through surgery and adjuvant therapy [21–23]. For Δ leu/PLT, the KM median-split showed borderline separation (*p* = 0.06), but the continuous per-SD Cox model—the prespecified primary analysis—was significant, and directionally concordant with Δ leu/PCT, supporting a consistent signal across leukocyte-weighted indices.

Concomitant temozolomide and greater adjuvant exposure were protective in our cohort, consistent with the survival advantage established by radiotherapy plus temozolomide and its adjuvant continuation in the landmark trial that defined modern standard of care [1].Clinically, these results argue for incorporating dynamic, CBC-based markers into peri-operative risk stratification and post-treatment surveillance, given their universality, low cost, and ease of serial sampling in routine care [5]. Monitoring leu/PCT is readily implementable because both inputs come from a routine complete blood count with differential and platelet indices. Most analyzers report PCT alongside PLT and MPV without extra blood draw or cost. However, we are aware that these measures can be platform-sensitive across analyzers and calibration practices. Thus reporting effects per + 1 SD enhances transportability across laboratories, but inter-platform variability remains a constraint, so multicenter validation with harmonized analyzer platforms and reference ranges is needed.

Several limitations should be acknowledged. First, this was a retrospective, single-centre analysis with limited sample size, which may restrict generalizability and introduce selection bias. Shorter survival likely reflects an older, frailer cohort with de-intensified therapy (fewer TMZ cycles; more hypofractionation). Age-related vulnerabilities and treatment interruptions further erode efficacy, aligning our outcomes with real-world series rather than trial populations enriched for fitter patients. Importantly, we lacked systematic capture of MGMT methylation. MGMT status remains one of the strongest established prognostic and predictive biomarkers in glioblastoma, particularly for response to temozolomide. The absence of this molecular variable limits our ability to disentangle whether the observed association between peri-operative hematologic dynamics—especially the Δ leukocyte-to-plateletcrit ratio—and overall survival might, in part, reflect underlying MGMT-related tumor biology. However, it is noteworthy that prior studies have shown peripheral inflammatory markers to retain prognostic relevance even when MGMT status is accounted for, suggesting that systemic host response contributes additional information beyond tumor methylation phenotype [24].

It is also well recognized that dexamethasone use in glioblastoma can alter leukocyte counts. Detailed preoperative dexamethasone dosing could not be ascertained for all patients, as many were referred from peripheral hospitals with limited data accessibility. At our institution, however, the perioperative corticosteroid regimen was standardized: patients typically received dexamethasone 8 mg three times daily for at least two days before surgery, continued at the same dose for two postoperative days, followed by a gradual taper. Consequently, individual cumulative doses could not be quantified and dose–response analyses were not feasible. The uniform protocol likely attenuates inter-patient variability in perioperative steroid exposure, but residual heterogeneity may persist, and generalizability to centers with different practices may be limited.

With 95 patients and multiple related ratios/deltas, the study is modestly powered and at risk of false positives despite family-wise BH-FDR control. We therefore emphasized effect sizes, directional consistency across related indices (Δ leu/PCT, Δ leu/PLT, reciprocal Δ PCT/leu), and bootstrap-validated discrimination; nevertheless, residual multiplicity and limited power warrant cautious interpretation and external validation. Finally, heterogeneity in radiotherapy fractionation (60/30 vs. 45/15) reflects real-world practice but may have introduced additional variability. These limitations highlight the need for prospective, multi-centre validation with comprehensive molecular annotation and standardized perioperative data collection together with proper comparison with existing prognostic models in glioblastoma.

In summary, we demonstrate that peri-operative increases in leukocytes relative to plateletcrit track independently with inferior OS in GB, complementing literature on inflammatory ratios and bringing a platelet mass–aware lens to hematologic prognostication in brain tumors. These data provide a rationale for validating leukocyte–PCT dynamics in multi-centre cohorts and for testing whether they improve established prognostic models or guide adaptive follow-up after standard chemoradiation.

## Conclusions

In GB, perioperative increases in leukocytes relative to plateletcrit (Δ leu/PCT) are independently associated with inferior overall survival, while concomitant temozolomide and greater adjuvant exposure are protective. Routine hematology trajectories are promising, low-cost prognostic candidates deserving prospective validation.

## Supplementary Information

Below is the link to the electronic supplementary material.

Supplementary Material 1

## Data Availability

Aggregate results are provided upon request.
