# Supplementary material for: Perioperative leukocyte–plateletcrit shift as a prognostic signature in glioblastoma
Source: J Neurooncol. 2025 Oct 20;176(1):27. doi: 10.1007/s11060-025-05302-8 (PMC12537585; doi:10.1007/s11060-025-05302-8)
Supplement: Supplementary file 1 — Supplementary Material 1 [file 11060_2025_5302_MOESM1_ESM.docx]

# Supplementary Methods and Results

## Δ-collinearity (post-operative − pre-operative)

Pairwise Spearman correlations were computed among Δ values and Δ ratios defined as post-operative minus pre-operative. Pairs with |r_s| ≥ 0.80 were considered highly collinear. In the leukocyte–platelet Δ family, Δ leu/PCT was chosen as the multivariable representative; Δ leu/PLT and Δ PCT/leu were excluded due to high collinearity.

### Table S1. Spearman correlation matrix for Δ metrics (post-op − pre-op).

|  | Δ leu | Δ PLT | Δ PCT | Δ MPV | Δ leu/PCT | Δ leu/PLT | Δ PCT/leu | Δ leu/MPV | Δ MPV/leu |
| --- | --- | --- | --- | --- | --- | --- | --- | --- | --- |
| Δ leu | 1.0 | 0.11 | 0.09 | 0.12 | 0.54 | 0.68 | -0.67 | 0.93 | -0.83 |
| Δ PLT | 0.11 | 1.0 | 0.96 | -0.12 | -0.42 | -0.36 | 0.43 | 0.09 | -0.09 |
| Δ PCT | 0.09 | 0.96 | 1.0 | -0.03 | -0.46 | -0.41 | 0.44 | 0.15 | -0.07 |
| Δ MPV | 0.12 | -0.12 | -0.03 | 1.0 | -0.11 | 0.06 | -0.12 | -0.13 | 0.07 |
| Δ leu/PCT | 0.54 | -0.42 | -0.46 | -0.11 | 1.0 | 0.96 | -0.62 | 0.52 | -0.45 |
| Δ leu/PLT | 0.68 | -0.36 | -0.41 | 0.06 | 0.96 | 1.0 | -0.66 | 0.65 | -0.6 |
| Δ PCT/leu | -0.67 | 0.43 | 0.44 | -0.12 | -0.62 | -0.66 | 1.0 | -0.6 | 0.75 |
| Δ leu/MPV | 0.93 | 0.09 | 0.15 | -0.13 | 0.52 | 0.65 | -0.6 | 1.0 | -0.86 |
| Δ MPV/leu | -0.83 | -0.09 | -0.07 | 0.07 | -0.45 | -0.6 | 0.75 | -0.86 | 1.0 |

### Table S2. Δ pairs with |r_s| ≥ 0.80 (post-op − pre-op).

| Δ metric A | Δ metric B | Spearman r_s | p-value |
| --- | --- | --- | --- |
| Δ PLT | Δ PCT | 0.96 | 4.43e-29 |
| Δ leu/PCT | Δ leu/PLT | 0.96 | 5.07e-29 |
| Δ leu | Δ leu/MPV | 0.93 | 1.25e-34 |
| Δ leu/MPV | Δ MPV/leu | -0.86 | 2.11e-24 |
| Δ leu | Δ MPV/leu | -0.83 | 1.29e-20 |
